# Neuroplastin in human cognition: review of literature and future perspectives

**DOI:** 10.1038/s41398-021-01509-1

**Published:** 2021-07-16

**Authors:** Katarina Ilic, Kristina Mlinac-Jerkovic, Goran Sedmak, Ivana Rosenzweig, Svjetlana Kalanj-Bognar

**Affiliations:** 1 Croatian Institute for Brain Research, School of Medicine, University of Zagreb, Šalata12, 10000 Zagreb, Croatia; 2 Sleep and Brain Plasticity Centre, Department of Neuroimaging, Institute of Psychiatry, Psychology and Neuroscience (IoPPN), King’s College London (KCL), Strand, London, WC2R 2LS UK; 3 Sleep Disorders Centre, Guy’s and St Thomas’ Hospital, Great Maze Pond, London, SE1 9RT UK

**Keywords:** Molecular neuroscience, Physiology, Hippocampus

## Abstract

Synaptic glycoprotein neuroplastin is involved in synaptic plasticity and complex molecular events underlying learning and memory. Studies in mice and rats suggest that neuroplastin is essential for cognition, as it is needed for long-term potentiation and associative memory formation. Recently, it was found that some of the effects of neuroplastin are related to regulation of calcium homeostasis through interactions with plasma membrane calcium ATPases. Neuroplastin is increasingly seen as a key factor in complex brain functions, but studies in humans remain scarce. Here we summarize present knowledge about neuroplastin in human tissues and argue its genetic association with cortical thickness, intelligence, schizophrenia, and autism; specific immunolocalization depicting hippocampal trisynaptic pathway; potential role in tissue compensatory response in neurodegeneration; and high, almost housekeeping, level of spatio-temporal gene expression in the human brain. We also propose that neuroplastin acts as a housekeeper of neuroplasticity, and that it may be considered as an important novel cognition-related molecule in humans. Several promising directions for future investigations are suggested, which may complete our understanding of neuroplastin actions in molecular basis of human cognition.

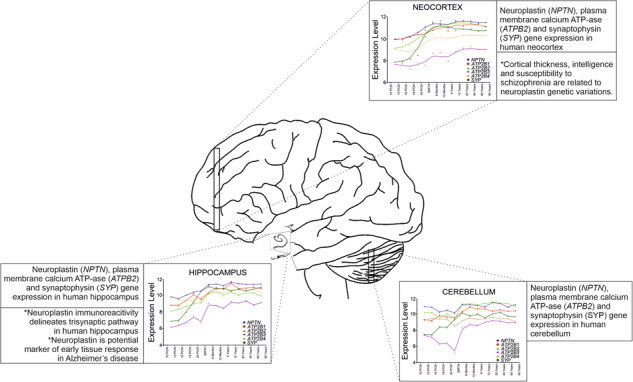

## Introduction

The quest for molecules crucially involved in molecular mechanisms of learning and memory has over the years been marred with limited success. To date, synaptic glycoprotein neuroplastin (Np), which belongs to a cell-adhesion molecule family, has been by and large marginalized in this quest. However, we propose that Np may be the very molecular candidate, which is linked specifically to memory formation and cognition in the human brain. Although important roles of Np in learning and memory have been demonstrated in rodents, its role in human physiology remains unclear and, so far, there has been no systematic attempt to collate and compare the available, albeit limited, data.

We here present the spatio-temporal gene expression of Np during the human lifespan, critically review the body of work on Np localization in the human central nervous system (CNS), and report all available published work to date on its expression in human tissues in different physiological and pathological conditions.

We further advocate its role in the CNS by discussing published genetic studies, which showed association of specific Np gene polymorphisms with cortical thickness and intellectual ability in adolescents, susceptibility to schizophrenia, and occurrence of neurodevelopmental disorders, e.g., autism [1–3]. We argue for its key role in the memory, by summarizing all available immunohistochemical data, which demonstrates specific immunolocalization of Np in adult human brain and in the trisynaptic hippocampal pathway [4, 5]. Role of Np in neurodegeneration is also suggested by demonstrating its involvement in tissue reorganization in Alzheimer’s disease (AD) [6], whereas its role in neurodevelopment and brain maturation is further argued by data analysis showing that the Np gene expression (*NPTN*) steadily increases from the prenatal period to adulthood when the expression is highest [7]. Finally, we hypothesize housekeeping role of Np in neuroplasticity, given its high level of expression in the CNS compared to other human brain genes in general.

We provide insight into exciting and promising perspectives of investigating synaptic protein Np as a novel human cognition-related molecule and highlight major discoveries related to its involvement in cognitive processes, from findings in rodents to most recent data with highest importance for the human brain.

## Neuroplastin discovery, structure, and functions

### Brief overview of Np research in mice and rats: from initial finding of a new synaptic protein to determining its roles in synaptic plasticity

During the last two decades, several pivotal studies in rodents and rodent cell cultures have suggested potential Np functions as follows: regulation of structure and function of synapses; regulation of excitatory and inhibitory synapses ratio; neurite outgrowth; long-term potentiation (LTP) regulation and maintenance of hippocampal CA1 synaptic plasticity; associative memory formation; cognitive deterioration; and tissue response following ischemic injury [1, 2, 8–18]. In-depth investigation of Np functions began with the characterization of two novel components of the brain synaptic subfraction, glycoproteins of *M*_r_ 65,000 and 55,000, named glycoprotein 65 and glycoprotein 55, which were eventually annotated as brain-specific Np65 and ubiquitous Np55 isoform, respectively [19]. (Different annotations of Np found in literature are summarized in Table 1.) Genetic and structural features of Np have been characterized [20–22] and its functions thoroughly investigated by in vivo and in vitro approaches [8, 13, 15–18].

**Table 1:** List of abbreviations, terms, and information related to mouse, rat, and human neuroplastin gene and protein isoforms, available in literature.

Neuroplastin gene names (Gene abbreviation; gene ID; chromosome)	Neuroplastin 55 isoform names (abbreviations)	Neuroplastin 65 isoform names (abbreviations)
Mouse (*Nptn*, *Sdrf1*, *Sdr1*; 20320; chr8) Rat (*Nptn*, *Sdrf1*, *Sdr1;*56064; chr9) Human (*NPTN*, *SDRF1*, *SDR1*; 27020; chr15)	Glycoprotein 55 (gp55) Neuroplastin 55 (Np55) Neuroplastin α (Nptnα; NPTNα) Stromal cell-derived factor receptor 1 (Sdfr1, SDFR1) Stromal cell-derived receptor 1 (Sdr1, SDR1) Stromal cell-derived receptor 1α (Sdr1α, SDR1α)	Glycoprotein 65 (gp65) Neuroplastin 65 (Np65) Neuroplastin β (Nptnβ; NPTNβ) Stromal cell-derived factor receptor 1 (Sdfr1, SDFR1) Stromal cell-derived receptor 1 (Sdr1, SDR1) Stromal cell-derived receptor 1β (Sdr1β, SDR1β)

Several studies indicate that Np, in partnership with basigin, exerts some of its effect(s) by acting as an obligatory subunit of the plasma membrane calcium ATPase (PMCA), therefore regulating Ca^2+^ ion homeostasis [5, 23, 24]. Recently, Np effects on spinogenesis and excitatory/inhibitory synapse ratio are found to be most likely regulated via tumor necrosis factor receptor-associated factor 6 (TRAF6) [25]. The adaptor protein TRAF6 is known to be involved in maintenance of the immune system homeostasis, through protein–protein interactions and cellular signaling linked to receptor families such as Toll-like receptors (TLRs) [26]. This finding suggests much higher complexity of potential Np functions in vital cellular processes than initially foreseen. Additional observations suggested that Nps are related to nervous cell energy metabolism and tissue response to ischemic injury as follows: acting as chaperones of monocarboxylate transporter MCT2 [14]; being involved in tissue response following ischemic injury in rat forebrain [10]; ensuring hypothesized mechanisms of tissue recovery as Np-knockout (KO) mice have been found to be more susceptible to ischemic brain lesions [27].

**Fig. 1:**
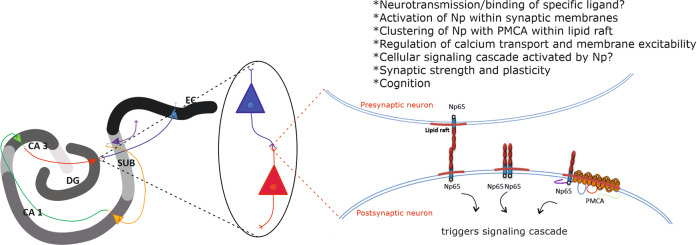
Hypothethical representation of functionally associated lipid raft residents—neuroplastin and plasma membrane calcium ATPase—and their involvement in molecular basis of cognition. Np, neuroplastin; PMCA, plasma membrane calcium ATPase; CA, Cornu Ammonis; DG, dentate gyrus; SUB, subiculum; EC, entorhinal cortex.

Further aspect of molecular complexity and machineries underlying processes related to memory, learning, and cognition encompasses so far a well-recognized influence of neuronal membrane composition, organization, and dynamics on synaptic plasticity [28, 29]. Our group reported that Np positioning within the neuronal membrane is highly sensitive on its particular membrane lipid environment in murine brain tissue [30]. Specifically, compositional changes of membrane glycosphingolipids–gangliosides alter Np gene and protein expression, as well its immunolocalization and distribution in the mouse hippocampus [30]. The finding that implies effects of membrane lipid composition on functionality of Np has been recently supported by a study describing specific rearrangements of Np and gangliosides upon chronic stress and aging in the rat hippocampus [31]. Most recently, Np has been shown to preferentially segregate within lipid rafts [32], neighboring with specific synaptic proteins that reside in rafts such as synaptophysin, GABA receptors, glutamate receptors, and PMCA [33]. Exact mechanism of action and signaling cascade triggered by Np is yet to be resolved; however, its intramembrane clustering with molecular partners involved in synaptic organization, neurotransmission, and ion homeostasis makes it at least an additional important factor in myriad of (patho)physiological events occurring on the neuronal cell surface. Undoubtedly, a research approach that combines investigation of cross-talk of various raft constituents involved in the regulation of membrane excitability, neurotransmission, and synaptic organization/plasticity, with an analysis of specific membrane lipid–protein interactions, should significantly contribute to understanding the complexity of molecular events related to cognition (Fig. 1).

### Np functions in rodent brain: observations from studies on mice with Np deficiency

An invaluable tool in investigating Np functions was the genetically engineered mouse not expressing Np [5, 16, 34, 35]. Data from studies of Np-deficient mice showed that constitutive Np KOs have altered hypothalamic-pituitary-adrenal axis correlates, elevated corticosterone levels, and decreased corticotropin-releasing hormone [16]. Although gross anatomy of the nervous system showed no differences compared to wild type, they displayed reduced motor capabilities and aberrant swimming behavior, less anxious behavior in open-field and light/dark avoidance tests, altered social interactions, and depressive-like behavior [16]. Ablation of Np in adult mice induced retrograde amnesia for associative learning [16]. In contrast, Amuti et al. [35] and Li et al. [36] reported that Np65 KO mice showed increased anxiety-like behavior, enhanced hippocampal-dependent memory enhanced sociability, and reduced depressive-like behavior, downregulated serotonin, and enhanced adult neurogenesis. Li et al. [36] suggest that contrasts in their results are due to different genetic deletions in those two mouse models. However, both models clearly show indispensable Np role in specific neuronal circuitry related to cognition.

### Np and regulation of intracellular calcium concentration

An important discovery of Np-interacting molecular partners and functions came from a study utilizing selective deletion of Np in glutamatergic neurons [5, 16]. The study revealed that neuronal Np is essential for the expression of PMCA, regulation of cytosolic Ca^2+^ levels, and normal circuit activity [5, 24]. Np is also expressed by cells of the immune system and it has been shown that Np-PMCA complexes are important modulators of T-cell activation [24].

Functional proteomic analysis confirmed that Np is indeed related to PMCA, by determining Np as the essential auxiliary subunit of PMCA [23]. PMCA is crucial in regulating cellular Ca^2+^ homeostasis in all eukaryotic cells [37]. Alternative splicing results in diversity of expression and localization of four PMCA paralogs (PMCA1–4) [38–41]. The human PMCA1 (hPMCA1)-Np complex has been structurally resolved by cryo-electron microscopy [37, 42]. Without Np, hPMCA1-alone proteins are devoid of ATPase activity and it has been determined that a transmembrane helix of Np interacts with specific transmembrane hPMCA1 regions [37]. Thus, through specific interactions, Np regulates Ca^2+^-binding site accessibility of PMCA [37].

Keeping in mind that Np has been implicated in LTP, which depends on Ca^2+^ homeostasis, findings that Np also functions as an obligatory subunit of PMCA and in that way affects intracellular Ca^2+^ concentration provide additional proof of the outmost importance of this protein for the molecular mechanisms governing human cognitive processes.

### Np in rodent model of obstructive sleep apnea: new perspectives for studying sleep and neuroplasticity in humans

The pivotal role of sleep in the regulation, modulation, and the promotion of neuroplasticity of the brain has gained significant appreciation over the last few decades [43]. Bidirectional links between both the macro- and micro-architecture of sleep and neuroplasticity have demonstrated its crucial role in the recovery of cognitive functions [44, 45]. In view of this fundamental function of sleep, perhaps unsurprisingly, our group has recently demonstrated Np’s involvement in recovery from one of the major sleep disorders, obstructive sleep apnea (OSA) [46], a condition linked to anxiety disorders, depression [47, 48], and AD [49–51], effectively with most of disorders that have so far also been linked with prominent Np plastic response. In our study of a rodent model of OSA, Np’s expression appeared to be dependent on the TLR2 functionality [46]. Arguably, this might be reflective of a more important role in the recovery process in the absence of TLR2 and/or of different mechanisms at play under control conditions. Either way, this finding debatably highlights the interlinking role for those two proteins.

In keeping with this emerging pattern of interlinking roles, the TLR2 receptors have been known to play an important role in AD pathogenesis [52]. Similarly, several recent pivotal studies suggested abnormalities in TLRs, including the TLR2 system, might be playing an important role in the pathophysiology of depression and suicidal behavior [53], stress-induced neuroinflammation [54], elevated anxiety, and social avoidance [55]. To successfully address any potential temporo-anatomical mechanics of when, where, who, and how of this possible tripartite relationship and the interplay between sleep, Np, and TLR2 system, future studies should be designed, which directly target the specific aspects of its purported cognitive and affective phenotypic aspects.

## Neuroplastin in humans: tissue expression, functions, and disorders

### Np immunoreactivity pattern in the human brain

The first study examining Np localization in the human brain showed a different pattern of immunohistochemical expression and distribution than in the rodent brain, explained to be related to species-specific functional diversity [4]. A more recent study by Herrera-Molina et al. [5] re-examined the data on Np65 immunohistochemical staining of selected human brain regions, with the main interest in human hippocampus, and analyzed the presence of Np isoforms in subcellular fractions derived from the human cortex. Not surprisingly, authors detected a much higher abundance of Np65 immunoreactivity in preparations of synaptic membranes, synaptic junctions, and postsynaptic densities compared to other subcellular fractions isolated from the human cortex. In the same study, Np65 immunoreactivity was found on neuronal cell bodies and fibers in the striatum (nucleus caudatus and putamen) and globus pallidus [5]. As stated before, Herrera-Molina et al. [5] were attentive to immunohistochemical expression and localization of Np in the human hippocampus and presented clear evidence of segregation of Np immunoreactivity in different hippocampal areas. More precisely, a distinct expression of Np was found in all major neuron-containing layers of the hippocampus—the stratum granulosum of the dentate gyrus (DG), the hilar part of the CA3 region, the pyramidal cell layer of CA1 and CA2-3, and the cellular layers of subiculum. Np65 immunoreactivity was also detected in the molecular layer of DG, a layer containing apical dendrites of granule cells. In addition, low Np65 immunoreactivity was detected in the stratum plexiforme and within the hilar neuropil of DG. The entorhinal cortex, a major hippocampal input region, also showed specific Np immunolocalization, most prominently on glutamatergic pyramidal neurons of layer II, IV, and V. On the cellular level, Np65 reactivity was reported on membranes of the cell bodies of granule cells and pyramidal neurons, dendrites, as well as in punctate structures within the neuropil [5]. Thus, immunoreactivity of brain-specific Np65 was found to specifically delineate the trisynaptic pathway, known as the principal excitatory hippocampal pathway. It is of note that CA1 and CA3 apical dendrites of the radiant and lacunar layer, as well as DG molecular and granular layer, are the targets of the perforant pathway composed of excitatory glutamatergic fibers. In addition, the synaptic output of the dentate granule cells includes formation of synapses with pyramidal neurons of the CA3 and the CA1—the specific connections involved in LTP. Axons of the pyramidal neurons of CA1 are considered to be the main hippocampal projections to subiculum and entorhinal cortex, and pyramidal layers of both subiculum and the entorhinal cortex show distinct Np signal. The layers known to be involved in internal and external regulatory circuits of the hippocampus–pyramidal cell layers of Cornu Ammonis (CA) and DG molecular layer containing interneurons show high and specific Np immunoreactivity [5]. Apart from the described expression of Np65 in the human hippocampus, it is clear that a much more detailed investigation of the cellular, regional, and temporal immunoreactivity pattern of Np in the human brain is needed. In addition, potential Np colocalization(s) with other known partners, which participate in molecular mechanisms underlying cognition and are clustered functionally within synaptic membranes, should be systematically examined. Not surprisingly, our unpublished observation indicates high immunofluorescence colocalization of Np and synaptophysin in major areas of adult human hippocampus (DG, CA1, and CA2/3), which corresponds with presumed functional association of these two synaptic proteins previously described in rat organotypic hippocampal slice cultures [56]. Apparently, utilization of modern high-resolution microscopy techniques may, in a short time, decipher the exact cellular and regional distribution of Np in the human brain. Such data along with findings of a great translational value from animal model studies will surely elucidate the extent and importance of Np actions in molecular events underlying human cognition.

### Spatio-temporal transcriptomic data for Np, PMCA, and synaptophysin in the human brain

**Fig. 2:**
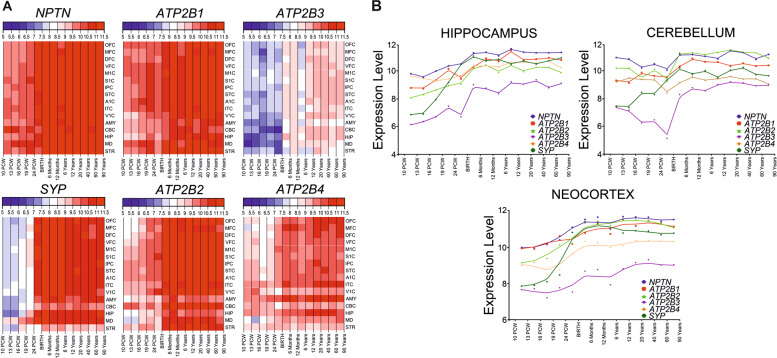
Spatio-temporal gene expression of neuroplastin, plasma membrane calcium ATPase and synaptophysin in human brain. **A** Spatio-temporal transcriptome of neuroplastin, plasma membrane calcium ATPase isoforms 1–4, and synaptophysin in the human brain as extrapolated from refs. [7, 58]. Heatmaps depict gene expression values (color code key at the top of heatmap), in different brain areas and developmental periods. **B** Comparison of gene expression values for neuroplastin, plasma membrane calcium ATPase isoforms and synaptophysin in the human neocortex, hippocampus, and cerebellum during development, adulthood, and aging. Developmental periods are shown on the *x*-axis and level of expression on the *y*-axis. A1C, primary auditory (A1) cortex; AMY, amygdala; *ATPB2[1–4]*, plasma membrane calcium ATPase isoforms 1–4; CBC, cerebellar cortex; DFC, dorsolateral prefrontal cortex; HIP, hippocampus; IPC, posterior inferior parietal cortex; ITC, inferior temporal cortex; M1C, primary motor (M1) cortex; MD, mediodorsal nucleus of the thalamus; MFC, medial prefrontal cortex; *NPTN*, neuroplastin; OFC, orbital prefrontal cortex; S1C, primary somatosensory (S1) cortex; STC, superior temporal cortex; STR, striatum; *SYP*, synaptophysin; V1C, primary visual (V1) cortex; VFC, ventrolateral prefrontal cortex.

Data on Np immunoreactivity pattern in the human brain are so far limited to the hippocampus and striatum. Our group has recently determined spatio-temporal gene expression of Np (*NPTN*) utilizing microarray database of healthy human brain (GEO accession GSE 25219, Human Exon 1.0 ST Array), as previously described [7]. Based on our observation on immunohistochemical colocalization of Np and synaptophysin in adult human hippocampus, and characterized functional interaction of Np and PMCA [23, 24], we have been interested as well in the expression pattern of genes coding for four PMCA isoforms and synaptophysin (*ATP2B1*, *ATP2B2*, *ATP2B3*, *ATP2B4*, and *SYP*). Transcriptomic analysis was performed in the hippocampus, cerebellum, and neocortex from 10 postconceptional weeks to 82 years of life, using a described method [57]. All six genes were expressed in all analyzed regions and time periods, and exhibited similar pattern of expression—lower levels of expression during prenatal period (Fig. 2A) and increase of expression during childhood, with the highest levels of expression during adulthood. The *NPTN* expression was higher than *ATP2B* and *SYP* in all analyzed brain regions and developmental periods (Fig. 2B). The only exceptions observed were *SYP* expression in the neocortex, hippocampus, and cerebellum, and *ATP2B2* expression in the cerebellum, reaching *NPTN* expression level at birth and mainly retaining the same expression level as *NPTN* during early childhood and adulthood (Fig. 2B). *ATP2B3* exhibited the lowest levels of expression in all analyzed regions and periods (Fig. 2B).

It is interesting to note that here the analyzed genes coding for Np, four PMCA isoforms, and synaptophysin exhibited very high levels of expression in adulthood compared to other genes in the human brain [7], even proportional to the expression of certain well-known housekeeping genes. Presented spatio-temporal transcriptomic analysis of synaptophysin expression pattern corresponds perfectly to the so far well-described functions of this synaptic protein in developing and adult human brain. The exact significance of the observed housekeeping level of particularly *NPTN* in practically all regions of the human brain has yet to be determined. We assume that Np gene expression pattern data strongly support its indispensable role in crucial cellular processes in the human nervous system, such as neural migration, synaptogenesis, neurotransmission, and establishing of neural circuitries.

### Np genetic variations and immunohistochemical expression in relationship with cognitive functions, neuropsychiatric disorders, and neurodegeneration

A study investigating the correlation of cortical thickness with intellectual ability in adolescents, as well as contribution of individual genes to those parameters, pinpointed Np as a protein whose single-nucleotide polymorphisms affect both [2]. Changes in cortical thickness in the left hemisphere have been found to correlate with performance of children on a test of general verbal intellectual functioning [58]. Comparison of estimates of genetic effects in the left and right hemispheres indicates that the language-dominant left cerebral cortex may be under stronger genetic control than the right cortex [59]. Association of one *NPTN* polymorphism (rs7171755) with effects on cortical thickness was found to be region-specific, with the most significant overall influences on the left temporal, frontal, and parietal lobes [2].

Following their publication on increased Np expression in rats after exposure to phencyclidine and methamphetamine, drugs known to induce long-lasting psychotic states [60], Saito et al. [1] investigated whether variations in Np gene confer genetic susceptibility to schizophrenia. In a study that included 200 patients with schizophrenia and 278 healthy volunteers, variations in SNPs in the 5′-upstream regions of *NPTN* were found, which may confer to genetic susceptibility to schizophrenia. In addition, a haplotype that was more frequently found in controls than patients with schizophrenia could be associated with mitigating the onset risk of schizophrenia, as its reduced transcriptional activity may interfere with excessive homophilic NPTN–NPTN interaction [1].

Furthermore, in a review by Smith et al. [3], Np is identified as a candidate implicated in the development of autism and neurodevelopmental disorders in general. The authors evaluate broad genetic mechanisms underlying genomic imbalances in autism, e.g., how would altered gene dosage and structure impact neurodevelopment and contribute to the risk for autism. The data regarding Np relies on an earlier study performed by the authors [61] in conjunction with two other studies from 2007 [62]. Taken together, the data show that Np was deleted in chromosomal microdeletion (15q24) in a patient with autism [61] and five patients exhibiting mild/moderate developmental delay [62, 63].

Genetic studies are a powerful tool in identifying underlying contributors to various complex pathological states. Therefore, these independent studies analyzing intellectual ability, schizophrenia, and autism, all pointing to Np, strongly indicate that Np should be considered in the evaluation of root causes of different neuropsychiatric disorders.

In addition to the cited genetic studies related to potential roles of Np in human neuropsychiatric disorders, there has been only one publication dealing with immunohistochemical analysis of Np expression in neuropathologically changed human brain tissue. The study was performed by our group—we analyzed immunohistological distribution of Np in human hippocampus affected by AD and reported increased Np65 immunoreactivity compared to age- and gender-matched samples in cases with disease duration shorter than 4 years. Np65 immunoreactivity was significantly increased in the DG, in particular on the dendrites of granule cells, pyramidal neurons of CA2/3, and subiculum. Changes in the expression were related to the progression of disease, as Np expression was decreased in all areas with prolonged duration of the disease (>5 years). We suggested that Np65 is involved in tissue reorganization and may be considered as a potential molecular marker of plasticity response in the early phase of AD pathological neurodegeneration cascade [6].

### Np expression and function in human non-neural tissues

Besides studies dealing with brain-specific Np isoform (Np65), findings from investigation of non-neural human tissues demonstrate that Nps are very likely involved in vital cellular processes such as cell proliferation and differentiation. Available data on Np expression in human tissues and different malignancies, summarized in Table 2, are supportive of suggested various actions of Np in human tissues according to previously published roles of other cell-adhesion molecules in intercellular interactions and communication [64–71]. Current knowledge on Np in rodents and humans implies actions potentially broader than the ones related to synaptic plasticity and these may be specifically associated with more ubiquitously expressed Np55 isoform. We propose that Np55 is involved in cellular housekeeping/“hardware” maintenance and participates in short-term cellular responses to numerous stimuli, whereas Np65 has specialized, sophisticated, cognition-related roles in differentiated neuronal populations. Thus, it will be of particular interest to search for, most probably, very diverse effects of each of the two Np isoforms in mammalian brain tissue but also their presumed cooperative actions in different patho(physiological) conditions.

**Table 2:** Neuroplastin in human tissues.

**Main finding**	**Detected on gene or protein level**	**Publication**
Neuroplastin is among the deregulated genes in mammary breast cancer models and in human breast cancer.	*NPTN* (*SDFR1*) gene overexpression	Rodriguez-Pinto et al. [67]
Higher neuroplastin expression may promote tumor invasion and/or metastatic potential in breast carcinomas.	NPTN (Npβ) protein overexpression (detected by proteomic identification of affinity selected tumor proteins)	Rodriguez-Pinto et al. [67]
Neuroplastin expression in human brain is detected in the cortex and cerebellum, but pattern differs from rodent brain.	Np protein expression (immunohistochemistry)	Bernstein et al. [4]
Single-nucleotide polymorphisms (SNPs) confer to genetic susceptibility to schizophrenia.	SNP in *NPTN* gene promoter region	Saito et al. [1]
Neuroplastin is deleted in chromosomal microdeletion and inverted duplication in a patient with autism and patients with developmental delay.	*NPTN* gene deletion	Filipek et al. [61], Sharp et al. [62], Klopocki et al. [63], Smith et al. [3]
Neuroplastin is upregulated in olfactory bulb-derived neural stem cells and human embryonic stem cells.	*NPTN* gene overexpression	Marei et al. [68]
Neuroplastin SNPs are linked to cortical thickness and intelligence in adolescents.	SNP in *NPTN* gene	Desrivieres et al. [2]
Neuroplastin colocalizes with extracellular matrix metalloproteinase inducer S100A8 and this interaction is important for keratinocyte proliferation (Np as S100A8/A9 receptor).	Np65 (Npβ) gene and protein expression investigated	Sakaguchi et al. [69]
Neuroplastin immunoreactivity delineates hippocampal sublayers that are involved in trisynaptic pathway.	Np65 protein expression (immunohistochemistry)	Herrera-Molina et al. [5]
Neuroplastin has the ability to induce cancer-related cellular events, such as growth, motility, and invasiveness in lung cancer cells as S100A8/A9 receptor.	Np65 (Npβ) gene and protein (detected by immunohistochemistry) expression investigated	Sumardika et al. [70], Tomonobu et al. [71]
Neuroplastin expression is higher in early stages of Alzheimer’s disease in hippocampus than in control hippocampi, and it decreases with the duration of the disease.	Np65 protein expression (immunohistochemistry)	Ilic et al. [6]

## Conclusions and future research prospects

Arguably, the complexity of molecular and cellular phenomena related to human cognition by far outstrips the effects of just one molecule. However, we propose that one molecule can make a significant difference—even in such a structurally intricate and functionally sophisticated system as the human brain. We suggest that synaptic protein Np should be considered as an important cognition-related molecule, which is supported by preliminary insights gained from here reported studies in the human brain. Np’s involvement in molecular mechanism of learning, memory, and cognition, has been undoubtedly confirmed in outlined studies in rodent brain and cell cultures. More recent data referring to Np gene and protein expression in the human brain strongly indicate its significant role in neuroplasticity and molecular events underlying cognitive processes, as well as its involvement in disorders accompanied with altered cognitive performance.

To more authoritatively ascertain the role for Np in the human brain, future studies will need to clarify the following: (1) structural features of Np in human tissues and probable effects of tissue- and species-specific glycosylation patterns on its functions; (2) membrane dynamics in physiological and pathophysiological conditions with emphasis on tracking the cross-talk of Np with other molecular partners within its specific (intra)membrane environment; (3) signaling mechanism of its cellular effects; (4) systematic mapping of its gene and protein expression in the human brain during critical periods of development, maturation, and aging; (5) cell types and neuronal circuitries marked by Np; (6) mechanisms underlying its expression in response to different (patho)physiological stimuli, related to its hypothesized role as a housekeeper of neuroplasticity.

From its initial discovery 30 years ago, Np has emerged as a likely significant factor involved in fine-tuning neuroplasticity and molecular events underlying cognitive processes in humans. We expect that Np research will be intensified in the future, shedding more light into the molecular mechanisms crucial for human cognition.
